# AMC: amyoplasia and distal arthrogryposis

**DOI:** 10.1007/s11832-015-0689-1

**Published:** 2015-11-04

**Authors:** Eva Kimber

**Affiliations:** Department of Pediatrics, Institute of Clinical Sciences at Sahlgrenska Academy, The Queen Silvia Children´s Hospital, Gothenburg, Sweden

**Keywords:** Arthrogryposis, Amyoplasia, Distal arthrogryposis, Muscle involvement, motor function, Contractures, Sarcomeric protein dysfunction

## Abstract

Arthrogryposis multiplex congenita (AMC) is a heterogeneous condition defined as multiple congenital joint contractures in two or more body areas. The common pathogenesis is impaired fetal movements. Amyoplasia, the most frequent form, is a sporadically occurring condition with hypoplastic muscles and joint contractures. Distal arthrogryposis (DA) syndromes are often hereditary, and joint involvement is predominantly in the hands and feet. In a Swedish study, 131 patients with arthrogryposis were investigated. The most frequent diagnoses were amyoplasia and DA. In amyoplasia, muscle strength was found to be more important than joint range of motion (ROM) for motor function. In DA, muscle weakness was present in 44 % of investigated patients. The clinical findings were found to be highly variable between families and also within families with DA. Fetal myopathy due to sarcomeric protein dysfunction can cause DA. An early multidisciplinary team evaluation of the child with arthrogryposis for specific diagnosis and planning of treatment is recommended. Attention should be directed at the development of muscle strength with early stimulation of active movements. Immobilization should be minimized.

## Introduction

Arthrogryposis multiplex congenita (AMC) is defined as congenital, non-progressive contractures in more than two joints and in multiple body areas. The term “multiple congenital contractures” may be used synonymously. The diagnosis is purely descriptive, and arthrogryposis can be present in at least 200 different syndromes [1]. The literature is confusing regarding types of arthrogryposis described, as different diagnoses are often lumped together and regarded as one entity.

The most frequently occurring form of arthrogryposis is amyoplasia [1], a sporadically occurring condition sometimes referred to as “classical arthrogryposis”. The second most common form is probably distal arthrogryposis (DA) [2, 3], a group of syndromes with mainly distal joint contractures [1, 4].

## Causes

Compromised fetal mobility is the main background factor, common to all different types of arthrogryposis. The cause can be pathology in the peripheral or central nervous system (CNS), in muscles or in connective tissue, defects in neuromuscular transmission, compromised space in utero, maternal disease, external factors like medication or drugs, or compromised vascular supply to the fetus [1].

It has been suggested that the joint fixations in multiple congenital contractures are caused by a proliferation of capsular connective tissue, a compensatory connective tissue response to lack of movement in utero [5].

Pathologic changes in children with AMC were described in a study by Banker in 1986 [6]. Abnormalities in the neuromuscular system were found in all cases, with the primary alterations in anterior horn cells, roots, peripheral nerves, motor end-plates, or muscles. All had onset during fetal development.

Investigation of the spinal cord in infants with neurogenic arthrogryposis has demonstrated abnormal histology and unequal distribution of alpha motor neurons in anterior horn cells, the latter being also predictive of involved muscle groups [7]. Several studies report that neurogenic arthrogryposis (of the amyoplasia type) can be caused by vascular compromise in early fetal development, with ischemia of anterior horn cells leading to fetal akinesia and poor or absent muscle development, but also, in some cases, to co-existing anomalies with presumed vascular genesis, i.e., gastroschisis, bowel atresia, Möbius syndrome, and focal muscle defects [1, 8–10].

Arthrogryposis can be present in several congenital myopathic disorders, e.g., nemaline myopathy [11], centronuclear myopathy [12], central core disease and others, in congenital muscle dystrophies (Fukuyama, Ullrich) [1], in severe SMA1 with fetal onset [13], and in severe congenital polyneuropathies [14–16].

Several DA syndromes have recently been discovered to be caused by mutations in sarcomeric muscle proteins [17–20].

A number of collagen disorders can also appear with arthrogryposis, for example Ehlers–Danlos syndrome [21], Marfan syndrome [22], and Larsen syndrome. DA9, Beals syndrome, has also been found to be a collagen disorder with a mutation in the fibrillin *FBN2* gene [23].

Disease affecting the neuromuscular junction with resulting weakness can cause arthrogryposis. It has been reported that congenital myasthenic syndrome with arthrogryposis [24], congenital myotonic dystrophy [25], and maternal antibodies to fetal neurotransmitters [26] can all cause arthrogryposis.

Movement restriction in utero caused by, e.g., oligohydramnios, myoma/fibroma of the uterus, and bicornate uterus can be associated with arthrogryposis. Arthrogryposis of the amyoplasia type occurs with increased frequency in one of monozygotic twins, but the cause in these cases is thought to be more related to vascular compromise than to actual crowding [8, 27].

A separate group of arthrogryposis syndromes are those caused by maternal disease during pregnancy, such as maternal multiple sclerosis, maternal myasthenia gravis, maternal diabetes mellitus, and maternal hyperthermia [1]. Metabolic disease such as phosphofructokinase deficiency can cause arthrogryposis, and drugs taken during pregnancy can also be associated with arthrogryposis (e.g., muscle relaxants, misoprostol, cocaine, alcohol) [1].

Contractures caused by compromised space in utero have a relatively late gestational onset and are relatively mild. These types of congenital contractures regress more easily compared to contractures caused by early immobility of the fetus [28].

## Occurrence

In a retrospective epidemiologic study in western Sweden by Darin et al., all children born with multiple congenital contractures between 1979 and 1994 were identified through screening of registers, reviews of medical records, and re-examination of children. Sixty-eight cases were identified, and the birth prevalence was found to be 1 in 5100 live births [29]. In this study, 39 patients had cerebral or spinal involvement, three patients had mechanical restriction in utero, 12 neuromuscular, and nine connective tissue involvement.

## Clinical classification

An approach to clinical evaluation that has been found to be useful has been suggested and further developed by Hall [1]. According to this, patients can be divided into three main groups of disorders: (1) primarily musculoskeletal involvement; (2) musculoskeletal involvement plus other system anomalies; and (3) musculoskeletal involvement plus CNS dysfunction and/or mental retardation (MR).

Amyoplasia or “classic arthrogryposis” belongs to the first group, as do several camptodactyly syndromes, DA type 1, popliteal pterygium syndrome, several symphalangism syndromes, and others.

In the second group, i.e., involvement of limbs and other body areas, several other camptodactyly syndromes, several DA syndromes, myotonic dystrophy, congenital myopathies, myasthenia gravis, connective tissue disorders such as Larsen syndrome, and Marfan syndrome with congenital contractures are included.

The third group includes a great number of genetic syndromes and chromosomal anomalies. Examples from this group are cerebro-oculo-facio-skeletal (COFS) syndrome, congenital muscular dystrophy, Miller–Dieker (lissencephaly), lethal multiple pterygium syndrome, Pena–Shokeir phenotype, Potter syndrome, Zellweger syndrome, trisomy 8/mosaicism, trisomy 18, and many others. This group includes lethal syndromes and syndromes with severe disabilities due to CNS malfunction.

Mental retardation/CNS involvement is found in approximately 25 % of individuals with AMC [1, 30].

Since there is a very large number of disorders with arthrogryposis, differential diagnosis can be difficult. It is, however, important to make as specific a diagnosis as possible for several reasons:

- Treatment can vary depending on the underlying cause in the individual child. For example, stretching should be minimized in diastrophic dysplasia, where too intensive mobilization may damage joint cartilage. The joint contractures can also be more resistant to treatment in certain types of AMC, especially amyoplasia [31], and surgery and splinting may need to be planned accordingly.
- Risk of re-occurrence varies greatly: amyoplasia occurs sporadically, while several forms of DA have autosomal dominant inheritance with a recurrence rate of 50 %.
- The prognosis is also very much dependent on diagnosis, where conditions with CNS involvement may have a poor prognosis, sometimes with early death, while other conditions have a normal lifespan but may need extensive orthopedic treatment and rehabilitation.

## Amyoplasia

The most common form of arthrogryposis is amyoplasia, which accounts for approximately one-third of all cases [1]. The word “amyoplasia” means no muscle formation. Amyoplasia occurs sporadically. Pathogenesis is unknown but thought to be impaired blood circulation to the fetus early in pregnancy, with hypotension and hypoxia damaging the anterior horn cells and resulting in lack of or underdevelopment of muscle tissue with fatty or connective tissue replacement [1, 32]. Clinically, the common morphologic features suggest a genetic syndrome, but occurrence is sporadic and individuals with amyoplasia have unaffected children [1]. Amyoplasia occurs in increased frequency in one of monozygotic twins [1].

The diagnostic criteria for amyoplasia are highly specific, with decreased muscle mass, typical joint contractures, and limb positioning at birth, mostly symmetrical in all four limbs. There may be involvement only of the lower limbs or, less commonly, only of the upper limbs and asymmetric limb involvement [1, 33]. Typically, the shoulders are adducted and internally rotated, and the elbows are extended with the forearms pronated and wrists and fingers flexed. The hips are either in abduction and external rotation with flexed knees, or flexed with extended or flexed knees. Hips and knees can be dislocated. The feet are most often in an equinovarus adductus position, although other types of foot deformities occur. Involvement of the spine is also described [1, 33]. The contractures can be fixed or flexible.

There is usually dimpling of the skin over affected joints. Common associated findings are midline facial hemangiomas and a round facial appearance. Muscle defects in the abdominal wall and inguinal hernias occur in about 10 % of children born with amyoplasia, and gastroschisis and bowel atresia have also been recorded [8]. Mental development is normal [33], unless there has been a concomitant birth asphyxia or other additional problems.

Contractures in children with amyoplasia are at their maximum at birth. To increase the joint range of motion (ROM) and to obtain a functional position of the joint, a combination of stretching, splinting, and orthopedic surgery is often necessary [34–36]. Early physical therapy is very important, both to mobilize joints and to stimulate muscle growth and to prevent further muscular atrophy [31]. In amyoplasia, the joint contractures can be severe and have a tendency to recur after correction [36].

## Distal arthrogryposis

DA syndromes are characterized by mainly distal congenital joint contractures, i.e., in the hands and feet. The exact incidence of DA is not known. In a large study of over 350 patients with arthrogryposis, 44 (12.6 %) patients with DA were identified [1]. In another review, 35 % of 155 patients with arthrogryposis were diagnosed with DA [3]. In an epidemiological survey from western Sweden, 5 (7 %) patients with DA syndromes were identified in a total of 68 patients with multiple congenital contractures [29].

DA has been defined as arthrogryposis with congenital hand and foot involvement by Hall et al. in 1982 [1]: type I with only distal joint involvement and characteristic hands at birth with flexed and overlapping fingers, and type IIA–IIE with distal limb contractures and additional characteristic manifestations.

A revised and extended classification was made by Bamshad et al. in 1996 [4]. In this classification, DA is defined as an inherited primary limb malformation disorder characterized by congenital contractures of two or more different body areas and without primary neurological and/or muscle disease that affects limb function. Included disorders are characterized by distal joint involvement, limited proximal joint involvement, autosomal dominant inheritance, reduced penetrance, and variable expressivity. In this classification, nine different clinical forms are originally described, with a tenth syndrome defined in 2006 [4, 37].

The prevalence of DA syndromes is not known. The most commonly described forms of DA are DA1 and DA2B. DA1 is characterized by clenched fists at birth, ulnar deviation, medially overlapping fingers, and club feet or other foot malpositions. The hips may be affected, calves small, and opening of the mouth mildly limited [38]. DA2B, Sheldon–Hall syndrome, is similar to but milder than DA2A [2, 39]. DA2A, Freeman–Sheldon syndrome, is characterized by a small mouth, facial contractures, scoliosis, mainly distal joint contractures, and short stature [39]. Typical findings in DA2B are vertical talus, ulnar deviation, severe camptodactyly, triangularly shaped face, prominent nasolabial folds, downslanting palpebral fissures, small mouth, and prominent chin [2]. Foot deformities may be asymmetric [40].

The sarcomere is the functional unit of striated muscle contraction. Mutations in sarcomeric proteins are found in at least 20 different skeletal muscle diseases [41] and can result in DA or in congenital myopathy [12].

Mutations in genes encoding sarcomeric muscle proteins are found in several DA syndromes: Sung et al. described mutations in β tropomyosin (*TPM2*), in DA1, and mutations in fast troponin I (*TNNI2*), and fast troponin T (*TNNT3*) in DA2B [17]. Toydemir et al. describes mutations in embryonic myosin heavy chain (*MYH3*) in DA2A, Freeman–Sheldon syndrome, and in DA2B, Sheldon–Hall syndrome [18]. Further, a mutation in fetal myosin heavy chain (*MYH8*) has been described in DA7, trismus-pseudocamptodactyly syndrome [19]. A defective function of contractile muscle proteins during fetal life influencing fetal mobility seems to be the common cause of congenital joint contractures in these syndromes [18, 19].

DA2A, Freeman–Sheldon syndrome, and DA2B, Sheldon–Hall syndrome, have been reported as the first disorders associated with mutations in embryonic MyHC (*MYH3*) [18]. DA syndromes are associated with missense mutations in various genes coding for sarcomeric proteins. The genes thus far demonstrated to be involved in DA syndromes are *TNNI2* (troponin I), *TPM2* (β-tropomyosin) [17], *TNNT3* (troponin T) [20], *MYH8* (perinatal MyHC), and *MYH3* (embryonic MyHC) [18].

These findings indicate that DA syndromes are caused by myopathies with onset during fetal development, but few studies have involved analysis of muscle tissue in these diseases.

## Swedish arthrogryposis study

In a nationwide study of arthrogryposis in children and adolescents in Sweden, 131 individuals were investigated [43]. Ages ranged from newborn to 28 years of age, with a mean age of 8.25 years. There were 59 females and 72 males. The majority of included individuals were under 20 years of age at the time of investigation.

Amyoplasia was diagnosed in 48 (37 %) patients; DA was found in 27 (21 %) index patients; 22 (17 %) children were found to have arthrogryposis with CNS involvement.

Six children had clinical signs of myopathy; different genetic syndromes were identified or suspected in ten children (Larsen syndrome, Turner syndrome, facio-audio-symphalangism syndrome Sprenger syndrome), six children had arthrogryposis in the lower extremities only, two of these with vertebral anomalies and caudal regression syndrome, and a further 12 children had arthrogryposis that we could not specify further.

Thirty-five patients with amyoplasia were studied separately [44]. Involvement of only the upper limbs was seen in four, only the lower limbs in six, and both the upper and lower limbs in 25 patients.

Strong correlations were found between muscle strength and motor function, and only moderate correlations between ROM and motor function.

Families with DA were also studied separately.

In one three-generation family with DA1 and a mutation in *TNNI2*, myopathy due to sarcomeric protein dysfunction was found to be the probable underlying cause [45].

In a mother and daughter with DA2B, muscle weakness associated with a *TPM2* mutation was found [46], and in patients from three families with *MYH3* mutations, developmental myosin myopathy with persistence postnatally was demonstrated [47].

Clinical and genetic findings in affected individuals with DA from 21 families were investigated, index cases and affected relatives [48].

A classification of the investigated patients with arthrogryposis into different specific diagnoses identified the three largest groups, in decreasing order, as amyoplasia, DA syndromes, and arthrogryposis with CNS involvement.

As a group, patients with amyoplasia have more severe muscle weakness and joint contractures than DA patients. Consequently, motor function is more compromised in amyoplasia. In DA syndromes, DA2A is more severe than DA2B, and DA1 is milder than DA2B.

In amyoplasia, muscle function is more important than severity of joint contractures for the prediction of walking ability and functional level. Joint position at birth is also of importance, especially for the prediction of ambulatory function. More attention should be paid to development of muscle strength with early stimulation of active movements. Immobilization should be minimized.

In DA1, 2A and 2B pathogenic mutations were found in *TNNI2*, *TPM2*, and *MYH3*. Troponin, tropomyosin, and myosin are sarcomeric proteins important for muscle contraction. These DA syndromes are caused by fetal myopathy due to sarcomeric protein dysfunction causing muscle weakness and secondary joint contractures.

DA1 and 2B are clinically and genetically heterogenous conditions. Mutations in the same gene were found in different DA syndromes, suggesting varying clinical penetrance and expression, rather than separate genetic syndromes. Different gene mutations were found in the same clinical syndrome, suggesting multiple gene background.

A pathogenic mutation is found more often in familial than in sporadically occurring cases, but de novo mutations are not uncommon.

DA syndromes may be re-defined according to pathogenic background, as several DA syndromes have been found to be caused by sarcomeric myopathy.

In studying the treatment of arthrogryposis, the main conclusions were that a careful clinical evaluation by a team with experience in arthrogryposis as early as possible is important for correct diagnosis and planning of treatment, and that care should be taken to minimize immobilization in order to avoid further muscle atrophy, especially during early life.

The majority of cases investigated in the study did not have a specific diagnosis other than arthrogryposis at the time of investigation. In some cases, further medical investigations such as muscle biopsy or radiology investigations were needed to clarify the diagnosis, but in many cases, careful clinical evaluation by professionals with experience in the evaluation of children with arthrogryposis was sufficient. Both in amyoplasia and in DA syndromes, diagnosis is based on clinical evaluation. An evaluation by a clinical geneticist is also invaluable in a number of cases, mainly those with specific malformation syndromes or dysmorphic features. A multidisciplinary evaluation by an experienced arthrogryposis team is needed for a correct diagnosis in the majority of cases.

In conclusion:

- A correct diagnosis is important.
- Early physical therapy, stretching of joint and muscle contractures, is important. The first 3–4 months of life are especially rewarding.
- Promote active muscle use—avoid immobilization.
- Functional assessment before surgery is important.
- Take care of pain management, avoid painful procedures.
